# Supplementary material for: Cortical and autonomic responses during staged Taoist meditation: Two distinct meditation strategies
Source: PLoS One. 2021 Dec 2;16(12):e0260626. doi: 10.1371/journal.pone.0260626 (PMC8638869; doi:10.1371/journal.pone.0260626)
Supplement: S1 Table — (PDF) [file pone.0260626.s001.pdf]

Table S1. Questionnaire data

Maria Volodina, Nikolai Smetanin, Mikhail Lebedev and Alexei Ossadtchi

variable	Novices	Meditators
age, years	35 (29-41)	35 (29-42)
BMI, kg/cm^2	23.8 (20.0-28.2)	22.2 (20.6-24.7)
waist to hip ratio	0.8 (0.7-0.9)	0.8 (0.7-0.9)
sex, males/females	9/6	5/8
years of meditative practice	0	8 (6-15)
sleep duration before experiment, hrs	7 (6-8)	7 (6-7)
usual sleep duration, hours	7 (7-8)	7 (6.5-7)
**before meditation**		
blood pressure, mmHg	111/79 (107/71-122/87)	116/72 (107/70 - 121/78)
HR, bpm	71 (62 - 84)	69 (66-72)
sleepiness, (1-10)	2 (1-3)	2 (1-3)
well-being, (1-10)	8 (8-10)	9 (8-9)
mood, (1-10)	9 (8-10)	8 (8-9)
anxiety, (1-10)	2 (1-2)	2 (1-2)
**after meditation**		
blood pressure, mmHg	111/79 (108/75 - 121/86)	111/74 (108/72-127/82)
HR, bpm	72 (62-78)	66 (63-70)
sleepiness, (1-10)	3 (1-5)	2 (2-5)
well-being, (1-10)	9 (8-10)	8 (8-8)
mood, (1-10)	9 (8-10)	8 (8-9)
anxiety, (1-10)	1 (1-2)	1 (1-2)
**difference of variable after and before meditation**		
blood pressure, mmHg	-1/-4 (-4/-3-3/5)	2/4 (-2/1-9/7)
HR, bpm *	0 (-7-2)	-1 (-4-0)
sleepiness, (1-10)	1 (0-2)	0 (0-1)
well-being, (1-10)	0 (0-1)	0 (-1-0)
mood, (1-10)	0 (0-0)	0 (-1-0)
anxiety, (1-10)	0 (-1-0)	0 (-1-0)

Data presented Median (IQR) where applicable. Novices (n = 15), meditators (n = 13) *- significant decrease after meditation in combined groups, paired t-test, p = 0.035
