# Supplementary material for: Cortical and autonomic responses during staged Taoist meditation: Two distinct meditation strategies
Source: PLoS One. 2021 Dec 2;16(12):e0260626. doi: 10.1371/journal.pone.0260626 (PMC8638869; doi:10.1371/journal.pone.0260626)
Supplement: S2 Table — (PDF) [file pone.0260626.s002.pdf]

Table S2. ANS activity markers during the resting state

Maria Volodina, Nikolai Smetanin, Mikhail Lebedev and Alexei Ossadtchi

variable	Novices	Meditators
**Heart rate variability**		
HR, bpm	76.0 (64.3-84.0)	72.3 (70.0-75.3)
min_RR, ms	653 (627-776)	712 (684-760)
max_RR, ms	930 (820-1081)	960 (898-985)
dRR, ms	193 (156-258)	210 (148-245)
RRNN, ms	802.6 (716.1-932.0)	822.6 (800.6-857.5)
SDNN,ms	51.8 (40.9-62.4)	42.6 (35.0-55.9)
CV,%	6.4 (4.4-7.6)	5.0 (4.3-6.5)
ME, ms	771.0 (714.5-923.0)	826 (793-866)
AME, %	50.0 (48.4-50.0)	48.6 (48.0-49.3)
RMSSD,ms	36.8 (25.1-49.9)	30.7 (24.6-33.9)
NN50	4 (1-9)	4 (2-6)
pNN50, %	8.22 (1.54-15.4)	5.8 (2.8-8.1)
SI, %/s^2	159.8 (115.7-181.3)	142.5 (117.8-211.4)
lf, ms^2	715.2 (166.5-1104.2)	427.2(213.6-762.6)
hf, ms^2	259.7 (93.2-493.4)	130.2 (88.0-278.9)
lf_nu	0.59 (0.48-0.9)	0.8 (0.48-0.9)
hf_nu	0.41 (0.11-0.5)	0.2 (0.1-0.52)
lf_peak, Hz	0.10 (0.08-0.1)	0.08 (0.07-0.1)
hf_peak, Hz*	0.17 (0.17-0.2)	0.20 (0.18-0.25)
lf/hf	1.4 (0.9-7.9)	3.9 (0.9-9.2)
volume pulse, a.u.	50412.8 (42896.3-108578.8)	51079.5 (38270.3-94843.3)
**Galvanic Skin Response**		
AUC, mV^2	0.07 (0.01-0.68)	0.03 (0.01-0.10)
Spontaneous reactions number	1.67 (0.67-4.67)	0.67 (0.00-2.67)
GSR, mV	-479.18 (-974.93- -192.03)	-313.96 (-371.28- -220.05)
**Respiration**		
respiration rate (per minute)	10.3 (8.3-12.0)	11.0 (9.0-13.7)
amplitude, mV	37.7 (21.2-54.8)	30.6 (22.2-57.9)

Data presented Median (IQR). Novices (n = 15), meditators (n = 13). * - significant difference (p<0.05), Mann-Whitney U test, U = 53,5, p = 0.045 SI - Baevsky's stress index (AME/(2*dRR*ME))
