# Supplementary material for: Cortical and autonomic responses during staged Taoist meditation: Two distinct meditation strategies
Source: PLoS One. 2021 Dec 2;16(12):e0260626. doi: 10.1371/journal.pone.0260626 (PMC8638869; doi:10.1371/journal.pone.0260626)
Supplement: S3 Table — (PDF) [file pone.0260626.s003.pdf]

Table S3. Comparison of ANS activity markers changes in the experienced meditators and the novices.

Maria Volodina, Nikolai Smetanin, Mikhail Lebedev and Alexei Ossadtchi

index	p-value
**Heart rate variability**	
HR	0,01
min_RR	0,39
max_RR	0
dRR	0
RRNN	0
SDNN	0
CV	0
ME	0
AME	0
RMSSD	0
SI	0
lf	0,01
hf	0,39
lf_nu	0,11
hf_nu	0,11
lf peak	0,92
hf peak	0
lf/hf	0,06
**Respiration**	
respiration rate	0
respiration amplitude	0
**GSR**	
Spontaneous reactions number	0,08
GSR	0,08

P-values according to AN-test (after FDR correction). P-values <0.05 marked with red
