# Supplementary material for: Cortical and autonomic responses during staged Taoist meditation: Two distinct meditation strategies
Source: PLoS One. 2021 Dec 2;16(12):e0260626. doi: 10.1371/journal.pone.0260626 (PMC8638869; doi:10.1371/journal.pone.0260626)
Supplement: S4 Table — (PDF) [file pone.0260626.s004.pdf]

Table S4. Changes of EEG-derived indices that had same dynamics in the both groups.

Maria Volodina, Nikolai Smetanin, Mikhail Lebedev and Alexei Ossadtchi

	resting state	stage 1	stage 2	stage 3	stage 4	stage 5
left parietal alpha	1	0.77(0.67-0.93)	0.69(0.54-0.86)** ##	0.70(0.54-0.91)*#	0.80(0.63-0.97)	0.86(0.69-1.20)
right parietal alpha	1	0.78(0.55-0.98)	0.57(0.46-0.99) ##	0.65(0.50-0.98) #	0.84(0.53-1.08)	0.89(0.66-1.37)
occipital alpha	1	0.80(0.57-0.99)	0.63(0.48-0.92)*##	0.68(0.53-0.91) #	0.77(0.54-1.07)	0.82(0.64-1.32)
left frontal beta	1	0.87(0.80-0.95)*	0.92(0.79-0.99) #	0,88(0.76-0.99)	0.95(0.77-1.06)	0.96(0.85-1.14)
right frontal beta	1	0.87(0.79-0.95) #	0.88(0.80-1.00)	0.86(0.78-0.98) #	0.95(0.75-1.07)	0.94(0.83-1.22)
frontal beta	1	0.88(0.80-0.99)	0.90(0.82-1.04) #	0.88(0.76-1.02) #	0.93(0.79-1.10) ##	0.94(0.87-1.19)
central beta	1	0.91(0.78-0.97)*#	0.87(0.77-0.96)*#	0.86(0.76-0.95)*##	0.89(0.80-1.04)	1.01(0.87-1.15)
parietal beta	1	0.86(0.75-0.96) ##	0.82(0.74-0.92)*##	0.83(0.74-0.96)*##	0.89(0.76-1.05) ##	1.04(0.91-1.21)
left temporal beta	1	0.83(0.75-0.95)**	0.88(0.75-0.93)**	0.87(0.77-0.95)**	0.89(0.76-0.93)	0.93(0.83-1.04)
left central beta	1	0.86(0.78-0.93)**	0.85(0.76-0.98)**	0.86(0.75-0.96)**	0.88(0.75-0.96)*	0.95(0.83-1.04)
left parietal beta	1	0.81(0.70-0.92)**	0.74(0.63-0.94)**#	0.76(0.62-0.86)**#	0.81(0.69-1.02)*	0.93(0.87-1.10)
right parietal beta	1	0,83(0.70-0.91)*	0.76(0.64-0.90)**#	0.84(0.61-0.89)**#	0.84(0.69-1.06)	1.02(0.78-1.17)
occipital beta	1	0.85(0.68-0.94)*	0.77(0.60-0.95)**	0.77(0.61-0.87)**#	0.83(0.66-1.04) #	1.02(0.82-1.10)

Changes of EEG-derived indices that had same dynamics in the both groups according to curve comparison test were analyzed using one-way ANOVA test. P-values were then corrected for multiple comparisons using the FDR. Ones that had significant effect of “meditation stage” factor were analyzed by post-hoc Tuckey test. Both groups together (n = 28) were analyzed. Data presented as Median ± IQR. *, ** - significant difference from resting state ($p<0.05$, $p<0.01$). #, ##- significant difference from 5th meditation stage ($p<0.05$, $p<0.01$).
