# Supplementary material for: Cortical and autonomic responses during staged Taoist meditation: Two distinct meditation strategies
Source: PLoS One. 2021 Dec 2;16(12):e0260626. doi: 10.1371/journal.pone.0260626 (PMC8638869; doi:10.1371/journal.pone.0260626)
Supplement: S5 Table — (PDF) [file pone.0260626.s005.pdf]

Table S5. Questionnaire data in “concentrated” and “relaxed” experienced meditators.

Maria Volodina, Nikolai Smetanin, Mikhail Lebedev and Alexei Ossadtchi

variable	"concentated"	"relaxed" meditators (n =
age, years	38(35-42)	29(29-50)
BMI, kg/cm^2	23.8(21.5-24.7)	21.6(19.8-22.9)
waist to hip ratio	0.83(0.8-0.86)	0.81(0.71-0.84)
sex, males/females	1/5	4/3
years of meditative practice	14(7-20)	8(4-12)
practice time, minutes per week	140(60-420)	210(120-420)
sleep duration before experiment, hrs	7(7-7)	6(6-7)
usual sleep duration, hours*	7(7-8)	6.5(5-7)
blood pressure, mmHg	115/71.5(113/70-121/72)	116/73(101/69-122/84)
HR, bpm	68(66-72)	71(65-73)
sleepiness, (1-10)	2.5(2-4)	2(1-3)
well-being, (1-10)	8.5(8-9)	9(8-9)
mood, (1-10)	8.5(8-10)	8(8-9)
anxiety, (1-10)	1.5(1-5)	2(1-2)

Data presented as Median (IQR). * - significant difference ($p<0.05$), t-test
