# Supplementary material for: Cortical and autonomic responses during staged Taoist meditation: Two distinct meditation strategies
Source: PLoS One. 2021 Dec 2;16(12):e0260626. doi: 10.1371/journal.pone.0260626 (PMC8638869; doi:10.1371/journal.pone.0260626)
Supplement: S6 Table — (PDF) [file pone.0260626.s006.pdf]

Table S6. Comparison of EEG-derived indices changes in the “relaxed” and “concentrated” experienced meditators and the novices.

Maria Volodina, Nikolai Smetanin, Mikhail Lebedev and Alexei Ossadtchi

index	p-value, relaxed vs concentrated	p-value, relaxed vs novices	p-value, concentrated vs novices
prefrontal delta	0	0,06	0,01
left frontal delta	0	0	0
right frontal delta	0,01	0,46	0,03
frontal delta	0	0	0,01
central delta	0	0	0,05
parietal delta	0,83	0,5	0,98
left temporal delta	0	0,22	0
right temporal delta	0,98	0,68	0,8
left central delta	0	0	0,38
right central delta	0	0,53	0,01
left parietal delta	0,98	0,86	0,95
right parietal delta	0,51	0,86	0,81
occipital delta	0,5	0,86	0,6
prefrontal theta	0,81	0,16	0,84
left frontal theta	1	0,16	0,45
right frontal theta	0,72	0,62	0,14
frontal theta	0,98	0,43	0,38
central theta	0,98	0,23	0,2
parietal theta	0,71	0,23	0,84
left temporal theta	0,89	0,07	0,13
right temporal theta	0,95	0,46	0,22
left central theta	0,98	0,11	0,1
right central theta	0,98	0,33	0,07
left parietal theta	0,5	0,32	0,98
right parietal theta	0,51	0,83	0,76
occipital theta	0,51	0,86	0,82
prefrontal alpha	0	0,02	0
left frontal alpha	0	0,07	0
right frontal alpha	0	0,02	0
frontal alpha	0	0,06	0

index	p-value, relaxed vs concentrated	p-value, relaxed vs novices	p-value, concentrated vs novices
central alpha	0	0,01	0
parietal alpha	0	0	0
left temporal alpha	0,03	0,44	0,1
right temporal alpha	0	0	0,08
left central alpha	0	0,28	0
right central alpha	0	0,03	0,01
left parietal alpha	0	0	0,13
right parietal alpha	0	0	0,01
occipital alpha	0	0	0,04
prefrontal beta	0,01	0,68	0,06
left frontal beta	0,02	0,03	0,6
right frontal beta	0	0,02	0,05
frontal beta	0,01	0,03	0,56
central beta	0,01	0,05	0,14
parietal beta	0	0	0,31
left temporal beta	1	0,08	0,65
right temporal beta	0,98	0,44	0,95
left central beta	0,14	0	0,84
right central beta	0,05	0,24	0,38
left parietal beta	0	0	0,7
right parietal beta	0,01	0,16	0,04
occipital beta	0	0	0,38
prefrontal gamma	0,98	0,6	0,4
left frontal gamma	0,98	0,96	0,86
right frontal gamma	0,57	0,8	0,04
frontal gamma	0,89	0,72	0,95
central gamma	0,98	0,86	0,71
parietal gamma	0,32	0,98	0,04
left temporal gamma	0,87	0,88	0,71
right temporal gamm	0,23	0,07	0,73
left central gamma	0,95	0,89	0,59

index	p-value, relaxed vs concentrated	p-value, relaxed vs novices	p-value, concentrated vs novices
right central gamma	0,98	0,76	0,46
left parietal gamma	0,38	0,24	0,71
right parietal gamma	0,9	0,98	0,54
occipital gamma	0,83	0,82	0,97
prefrontal alpha to theta	0	0,59	0
left frontal alpha to theta	0	0,82	0
right frontal alpha to theta	0	0,19	0
frontal alpha to theta	0	0,27	0
central alpha to theta	0	0,07	0
parietal alpha to theta	0	0,08	0
left temporal alpha to theta	0	0,49	0
right temporal alpha to theta	0	0,02	0
left central alpha to theta	0	0,76	0
right central alpha to theta	0	0,11	0
left parietal alpha to theta	0	0	0,01
right parietal alpha to theta	0	0	0,05
occipital alpha to theta	0	0	0,22
prefrontal alpha to beta	0	0,5	0
left frontal alpha to beta	0	0,98	0
right frontal alpha to beta	0	0,89	0
frontal alpha to beta	0	0,98	0
central alpha to beta	0	0,63	0
parietal alpha to beta	0	0,08	0
left temporal alpha to beta	0	0,8	0
right temporal alpha to beta	0	0,11	0
left central alpha to beta	0	0,84	0
right central alpha to beta	0	0,59	0
left parietal alpha to beta	0	0,43	0
right parietal alpha to beta	0	0,01	0
occipital alpha to beta	0	0,01	0

FDR corrected p-values according to AN-test. P-value<0.05 marked with red
