# Supplementary material for: Cortical and autonomic responses during staged Taoist meditation: Two distinct meditation strategies
Source: PLoS One. 2021 Dec 2;16(12):e0260626. doi: 10.1371/journal.pone.0260626 (PMC8638869; doi:10.1371/journal.pone.0260626)
Supplement: S8 Table — (PDF) [file pone.0260626.s008.pdf]

Table S7. Comparison of ANS activity markers changes in the "relaxed" and "concentrated" experienced meditators and the novices.

Maria Volodina, Nikolai Smetanin, Mikhail Lebedev and Alexei Ossadtchi

index	p-value, relaxed vs concentrated	p-value, relaxed vs novices	p-value, concentrated vs novices
HR	0,00	0,97	0,00
min_RR	0,00	0,00	0,02
max_RR	0,64	0,01	0,00
dRR	0,00	0,00	0,25
RRNN	0,00	0,97	0,00
SDNN	0,00	0,00	0,07
CV	0,00	0,00	0,37
ME	0,00	0,92	0,00
AME	0,84	0,00	0,00
RMSSD	0,29	0,00	0,05
SI	0,00	0,00	0,02
lf	0,00	0,00	0,97
hf	0,17	0,82	0,37
lf_nu	0,00	0,01	0,56
hf_nu	0,00	0,00	0,97
lf peak	0,84	0,92	0,97
hf peak	0,65	0,00	0,00
lf/hf	0,00	0,00	0,48
respiration rate	0,02	0,00	0,02
respiration amplitude	0,00	0,00	0,06
AUC (GSR)	0,84	0,21	0,18
Spontaneous reactions number	0,54	0,02	0,41
GSR	0,96	0,22	0,37

FDR corrected p-values according to AN-test. p-value<0.05 marked with red
